# Sensor Fault Detection and System Reconfiguration for DC-DC Boost Converter

**DOI:** 10.3390/s18051375

**Published:** 2018-04-28

**Authors:** Jian Li, Zhigang Zhang, Bo Li

**Affiliations:** School of Automation Engineering, Northeast Electric Power University, Jilin 132012, China; lijian@neepu.edu.cn (J.L.); zhangzhigangyx@outlook.com (Z.Z.)

**Keywords:** sensor fault, boost converter, Luenberger observer, fault detection, system reconfiguration

## Abstract

This paper presents a effective sensor fault detection and system reconfiguration approach for DC-DC Boost converter. We consider to design a Luenberger observer to solve the problem of the sensor fault detection of the DC-DC Boost converter. We establish mathematical model according to the state of the switch . Luenberger observer is designed to produce residual errors and analyse faults. We detect three types of sensor faults online and compare residuals with thresholds. The system is able to maintain stability by taking system reconfiguration approach. The effectiveness of this approach is demonstrated by simulations.

## 1. Introduction

In recent decades, sensor fault detection in power electronics has aroused increasing attention. Power electronics products, such as switching power supply, frequency converter and UPS power supply, have been widely used in all aspects of social life and national economy such as aerospace, automobile system, vehicle system, ship system, medical equipment and so on [1,2]. The safe operation of power electronic products is of great significance to the safety, efficiency and quality of the entire system.

According to the power conversion type of the power treatment device, The switching converters have the following four basic forms:AC-DC converters, AC-AC converters, DC-AC converters, DC-DC converters. Thereinto, DC-DC converters have widely applied to many fields, including multiple input source applications [3], offshore wind power [4] and so on. To achieve an ultrahigh step-up ratio while maintaining a high conversion efficiency, a novel DC-DC converter topology is proposed in [5]. The design and control of DC-DC converters is an important branch of electronic technology. There are three common fault in DC-DC converters: open -circuit fault, gain deviation fault and noise abnormity fault. In this case, some solutions about fault detection and fault tolerant control methods have been proposed [6,7,8,9]. In [10], the model-based estimator approach is used to design a fault diagnosis method for switching power converters . In [11], based on analytical redundancy relations, the paper design the sensor fault detection and isolation method. It is the role of transforming the direct current of input into the direct current of output which has different characteristics. Thereinto, the DC-DC boost converter is widely used into daily life. This is a direct-current converter with an output voltage higher than the input voltage. In recent years, some papers propose different methods to research and analysis this boost converter [12,13,14,15,16,17,18]. In [19], the paper proposed a open circuit switch fault detection method which is very fast and effective with the fault tolerant converter topology.

In the field of automatic control, sensors are the main devices for information acquisition. When a sensor faults occurs, it will have a serious impact on the follow-up monitoring and controlling. Therefore, the researching of sensor fault detection is particularly important. Under these circumstances, some papers proposed different sensor fault detection methods [20,21,22,23]. In addition, there are some reconfiguration schemes for fault tolerant control [24,25,26,27] . Common sensor faults mainly include three kinds of faults: open-circuit fault, gain deviation fault and noise abnormity fault. Real-time detection of DC-DC converter fault by online and the appropriate fault-tolerant control approaches can improve the fault-tolerant ability of the system .

Many kinds of observer-based fault detection methods have been proposed which generate residuals to detect system fault information [28,29,30,31]. More recently, different kinds of observers have been used for fault detection, including Luenberger observers, adaptive observers, sliding mode observers and so on. To observe the states and estimate the component parameters , the paper designs a model-observer-based scheme for Buck converters [32]. The Luenberger observers are able to estimate current and voltage accurately. In [33], the paper design a Luenberger observer for the sensor fault detection and fault tolerant control.

This paper propose a sensor fault detection and system reconfiguration method for the DC-DC Boost converter. Firstly, the boost converter is described as a switched model, which can precisely capture the characteristics between different working modes of the converter. Secondly, a fault detection observer is designed based on the faulted mode to construct residual signal. The residual value is larger than the threshold when the sensor fault happens, an alarm is generated. Thirdly, we design a switching control law for the Boost converter under healthy condition. Then we put to use a system reconfiguration method to improve the fault-tolerant ability of the system. Finally, we apply the method proposed above to three kinds of sensor faults in the current sensor and the voltage sensor.

The structure of the paper is organized as follows. The mathematical model of the Boost converter is established in Section 2. The Luenberger observer is designed in Section 3. The switching control law for healthy condition is presented in Section 4. In Section 5, we present fault detection and system reconfiguration scheme design. In Section 6, The efficiency of this approach is demonstrated by simulation examples. Finally, we arrive at the conclusion of this paper in Section 7.

## 2. System Model

**Figure 1:**
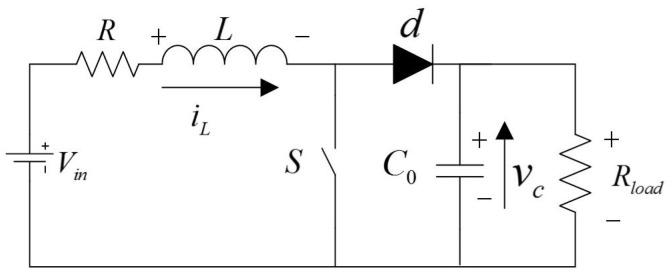
Schematic representation of the DC-DC Boost converter.

The DC-DC Boost converter circuit is shown in Figure 1. The circuit is able to get a higher voltage through the switch *S* . This process contains two operating modes. The switch is open (*S* = 0) when the system is in mode 1. The switch is closed (*S* = 1) in mode 2 . The two operating modes can be described as follows :

$$\left\{\begin{array}{cc} S & =0\\ \frac{d}{dt}i_{L} & =-\frac{R}{L}i_{L}-\frac{1}{L}v_{c}+\frac{1}{L}V_{in}\\ \frac{d}{dt}v_{c} & =\frac{1}{C_{0}}i_{L}-\frac{1}{R_{load}C_{0}}v_{c} \end{array}\right.\;\left\{\begin{array}{cc} S & =1\\ \frac{d}{dt}i_{L} & =-\frac{R}{L}i_{L}+\frac{1}{L}V_{in}\\ \frac{d}{dt}v_{c} & =-\frac{1}{R_{load}C_{0}}v_{c} \end{array}\right.$$

Then we can get the mathematical model of the circuit:

(1) $$\begin{array}{cc} \dot{x}\left(t\right) & =A_{\sigma (t)}x\left(t\right)+Bu\left(t\right)\\ y(t) & =Cx(t) \end{array}$$

In the above equation, x(t) is the state vector, y(t) is the output vector, u(t) is the input vector, σ(t) appears the switching signal generated by converter control. $A_{\sigma (t)}$, *B* and *C* are the collection of state space models. Where x(t) , $A_{\sigma (t)}$, *B* and *C* are set as:

$$x\left(t\right)=\left[\begin{array}{c} i_{L}\left(t\right)\\ v_{c}\left(t\right) \end{array}\right],A_{\sigma (t)}=\left[\begin{array}{cc} -\frac{R}{L} & -\frac{1-S}{L}\\ \frac{1-S}{C_{0}} & -\frac{1}{R_{load}C_{0}} \end{array}\right],B=\left[\begin{array}{c} \frac{1}{L}\\ 0 \end{array}\right],C=\left[\begin{array}{cc} 1 & 0\\ 0 & 1 \end{array}\right].$$

**Remark** **1.** *In this paper, two state vector $\left(i_{L},v_{c}\right)$ is used to build the system model and analyse the mathematical model. Because we hope to get a stable output voltage for DC-DC boost converter. The voltage of capacitor is equal to output voltage. Therefore, we focus on the state vector $v_{c}$.*

**Figure 2:**
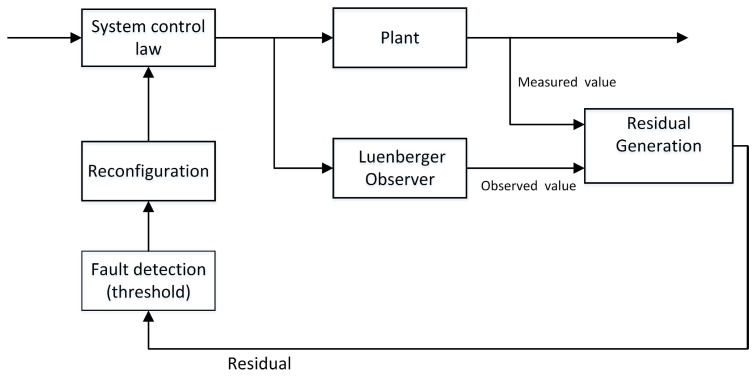
Block scheme of the fault detection and system reconfiguration.

In the next sections, we need to realize the sensor fault detection and the system reconfiguration. We design a Luenberger observer to produce a residual signal and compare it with a predefined threshold. When the sensor fault happens, the residual value is larger than the threshold and an alarm is generated. Thus, the value of Luenberger observer is applied to system switching law instead of measured value. In addition, the output value *y* is replaced by the desired value $x^{*}$. Consequently, The system is able to maintain stability by taking system reconfiguration approach and we can obtain a stable output voltage. The block scheme of the fault detection and system reconfiguration is shown in Figure 2.

## 3. Luenberger Observer Design

Since sensor faults appear in the system, the output value y(t) will have some changes. So we can describe the fault model of system as:

(2) $$\begin{array}{cc} \dot{x}\left(t\right) & =A_{\sigma (t)}x\left(t\right)+Bu\left(t\right)\\ y(t) & =Cx(t)+Qf(t) \end{array}$$

where Q=I with appropriate dimensions and f(t) is the sensor fault signal which caused by the undesirable sensor faults in the electric circuit. In order to realize fault detection, the following Luenberger observer for the switched system is constructed:

(3) $$\begin{array}{cc} \dot{\hat{x}}\left(t\right) & =A_{\sigma (t)}\hat{x}\left(t\right)+Bu\left(t\right)+H\left(y\left(t\right)-\hat{y}\left(t\right)\right)\\ \hat{y}\left(t\right) & =C\hat{x}\left(t\right)\\ r(t) & =y\left(t\right)-\hat{y}\left(t\right) \end{array}$$

where $\hat{x}\left(t\right)$ is the observer state, $\hat{y}\left(t\right)$ is the observer output, *H* is the observer gain matrix and r(t) is the observer residual vector .

Denoting $e\left(t\right)=x\left(t\right)-\hat{x}\left(t\right)$, the augmented state vector $\tilde{x}\left(t\right)=e\left(t\right)$, $\tilde{r}\left(t\right)=r\left(t\right)-f\left(t\right)$. The augmented system of (2) and (3) can be written as

(4) $$\begin{array}{c} \dot{\tilde{x}}\left(t\right)=\tilde{A}\tilde{x}\left(t\right)+\tilde{B}f\left(t\right)\\ \tilde{r}\left(t\right)=\tilde{C}\tilde{x}\left(t\right)+\tilde{D}f\left(t\right) \end{array}$$

where

(5) $$\begin{array}{c} \left[\begin{array}{cc} \tilde{A} & \tilde{B}\\ \tilde{C} & \tilde{D} \end{array}\right]=\left[\begin{array}{cc} A_{\sigma (t)}-HC & -H\\ C & 0 \end{array}\right] \end{array}$$

The frameworks of fault detection observer design: the Luenberger state-observer is designed such that the augmented system (4) is asymptotically stable when f(t)=0 and under zero-initial condition, For detection objective, the effects from the faults to the residual error signal $\tilde{r}\left(t\right)$ are minimized. The fault detection observer satisfies the following index :

(6) $$\begin{array}{c} \int_{0}^{\infty }{\tilde{r}}^{T}\left(t\right)\tilde{r}\left(t\right)dt<\gamma^{2}\int_{0}^{\infty }f^{T}\left(t\right)f\left(t\right)dt \end{array}$$

In the following theorem, we obtain the necessary condition that system (4) satisfies the upper requirement. The observer gain *H* can be achieved at the same time.

**Theorem** **1.** Given the constant $a_{1\sigma }$ , if there exist matrix variables W, H, $P=\left[\begin{array}{cc} P_{1} & P_{2}\\ {}* & P_{3} \end{array}\right]>0$ satisfying the inequalities

(7) $$\begin{array}{c} \left[\begin{array}{cccc} -W-W^{T} & P+W^{T}A_{\sigma }-HC-a_{1\sigma }W & -H & 0\\ {}* & a_{1\sigma }W^{T}A_{\sigma }+a_{1\sigma }A_{\sigma }^{T}W-a_{1\sigma }HC-a_{1\sigma }C^{T}H^{T} & -a_{1\sigma }H & C^{T}\\ {}* & * & -\gamma^{2}I & 0\\ {}* & * & * & -I \end{array}\right]<0 \end{array}$$

*then the system (4) under arbitrary switching is asymptotically stable, and guarantees the robust performance (6). Moveover, if (7) is feasible, then the observer gain in form of (3) can be given by $H={\left(W^{T}\right)}^{-1}H$.*

**Proof** **of Theorem 1.** Firstly, consider the stability for the system (4), we rewrite the system as $\dot{\tilde{x}}\left(t\right)=\tilde{A}\tilde{x}\left(t\right)$ when f(t)=0, and choose the common Lyapunov functions: $V\left(\tilde{x}\left(t\right)\right)={\tilde{x}}^{T}\left(t\right)P\tilde{x}\left(t\right).$ Then it has

(8) $$\begin{array}{c} {\dot{V}}_{\sigma }\left(\tilde{x}\left(t\right)\right)={\tilde{x}}^{T}\left(t\right)\left({\tilde{A}}^{T}P+P\tilde{A}\right)\tilde{x} \end{array}$$

We consider the following performance index $\Gamma \left(t\right)=\int_{0}^{t}\left({\tilde{r}}^{T}\left(\tau \right)\tilde{r}\left(\tau \right)-\gamma^{2}f^{T}\left(\tau \right)f\left(\tau \right)\right)d\tau$. For any nonzero $f\left(t\right)\in l_{2}\left[\left.0,\infty \right)\right.$ and the under zero-initial condition, we have

$$\begin{array}{cc} \Gamma (t) & =\int_{0}^{t}\left({\tilde{r}}^{T}\left(\tau \right)\tilde{r}\left(\tau \right)-\gamma^{2}f^{T}\left(\tau \right)f\left(\tau \right)+{\dot{V}}_{\sigma }\left(\tilde{x}\left(\tau \right)\right)\right)d\tau -V_{\sigma }\left(\tilde{x}\left(t\right)\right)\\ & \leq \int_{0}^{t}\left({\tilde{r}}^{T}\left(t\right)\tilde{r}\left(t\right)-\gamma^{2}f^{T}\left(t\right)f\left(t\right)+{\dot{V}}_{\sigma }\left(\tilde{x}\left(\tau \right)\right)\right)d\tau \end{array}$$

It notes that

$$\begin{array}{c} {\tilde{r}}^{T}\left(\tau \right)\tilde{r}\left(\tau \right)-\gamma^{2}f^{T}\left(\tau \right)f\left(\tau \right)+{\dot{V}}_{\sigma }\left(\tilde{x}\left(\tau \right)\right)={\left[\begin{array}{c} \tilde{x}\left(\tau \right)\\ f(\tau ) \end{array}\right]}^{T}\Lambda \left[\begin{array}{c} \tilde{x}\left(\tau \right)\\ f(\tau ) \end{array}\right] \end{array}$$

where

$$\begin{array}{c} \Lambda =\left[\begin{array}{cc} {\tilde{A}}^{T}P+P\tilde{A}+{\tilde{C}}^{T}\tilde{C} & P\tilde{B}+{\tilde{C}}^{T}\tilde{D}\\ {}* & -\gamma^{2}I+{\tilde{D}}^{T}\tilde{D} \end{array}\right] \end{array}$$

Thus, if Λ<0, it follows form (8) that ${\dot{V}}_{\sigma }\left(\tilde{x}\left(t\right)\right)<0$,which implies that $V(\tilde{x}\left(t\right))$ converges to zero as t→∞. The switched system (4) under arbitrary switching is asymptotically stable. Moreover, it also implies Γ(t)<0. Then, it has the robust performance (6).

It notes that Λ can be rewritten as

(9) $$\begin{array}{c} \left[\begin{array}{ccc} {\tilde{A}}^{T} & I & 0\\ {\tilde{B}}^{T} & 0 & I \end{array}\right]\left[\begin{array}{ccc} 0 & P & 0\\ P & {\tilde{C}}^{T}\tilde{C} & {\tilde{C}}^{T}\tilde{D}\\ 0 & {\tilde{D}}^{T}\tilde{C} & -\gamma^{2}I+{\tilde{D}}^{T}\tilde{D} \end{array}\right]\left[\begin{array}{cc} \tilde{A} & \tilde{B}\\ I & 0\\ 0 & I \end{array}\right]<0 \end{array}$$

By Projection theorem, (9) is equivalent to

(10) $$\begin{array}{c} \left[\begin{array}{ccc} 0 & P & 0\\ P & {\tilde{C}}^{T}\tilde{C} & {\tilde{C}}^{T}\tilde{D}\\ 0 & {\tilde{D}}^{T}\tilde{C} & -\gamma^{2}I+{\tilde{D}}^{T}\tilde{D} \end{array}\right]+He\left(\left[\begin{array}{c} -I\\ {\tilde{A}}^{T}\\ {\tilde{B}}^{T}\; \end{array}\right]\left[\begin{array}{ccc} W & a_{1\sigma }W & 0 \end{array}\right]\right)<0 \end{array}$$

where *W* is the matrix variables of appropriate dimensions. By denoting $H=W^{T}H$ and applying schur complement formula, and after some matrix manipulation, (10) becomes (7). ☐

**Remark** **2.** *Theorem 1 has formulated the inequality conditions for the stability and the performance (6). As long as the parameters satisfy the inequalities, the system is asymptotically stable and satisfies the robust performance index (6). Moreover, it is noted that condition (7) is all convex. Hence, the problem of observer design can be directly translated into solve (7). The observer gain can be derived by $H={\left(W^{T}\right)}^{-1}H$.*

## 4. System Control Law

### 4.1. Design of Control Law

In [34], the paper design a switching control law for the Boost converter under healthy condition. In this paper, we use this switching law to guarantee the stability of the system. In this process, we will use a Lyapunov function, see [35]. Denote $z=x-x^{*}$, then we consider the system

(11) $$\begin{array}{c} \dot{z}\left(t\right)=A_{\sigma }z\left(t\right)+A_{\sigma }x^{*}+Bu\left(t\right) \end{array}$$

where $x^{*}={\left(i_{L}^{*},v_{c}^{*}\right)}^{T}$. Moreover, $i_{L}^{*}>0$ and $v_{c}^{*}>0$ are desired current and voltage. We choose the control Lyapunov function

(12) $$\begin{array}{c} V\left(x\right)=z^{T}Pz \end{array}$$

$P=\left[\begin{array}{cc} p_{11} & 0\\ 0 & p_{22} \end{array}\right]>0$ . Since S∈{0,1} represents two modes of operation, there exist two derivatives ${\dot{V}}_{0}$(x) {S = 0} and ${\dot{V}}_{1}$(x) {S = 1}. The switching law of system (1) is designed as follows:

(1) If ${\dot{V}}_{0}\left(x\right)<{\dot{V}}_{1}\left(x\right)$, the system is in mode 1, in which the switch is open (S = 0).
(2) If ${\dot{V}}_{1}\left(x\right)<{\dot{V}}_{0}\left(x\right)$, the system is in mode 2, in which the switch is closed (S = 1).

**Remark** **3.** *For guaranteeing the switching system (11) is asymptotically stable, we consider $\dot{V}\left(x\right)<0$. For Lyapunov function V(x), the derivative of V(x) is a negative number. So the smaller the derivative, the greater the attenuation rate of V(x). This condition is used to derive the upper switching law. Therefore, we choose the switching law as follows:*

(13) $$\begin{array}{c} \sigma \left(x\left(t\right)\right)=\underset{\sigma }{\arg\min}\left\{{\dot{V}}_{\sigma }\left(x\right)<0\right\}. \end{array}$$

By taking this switching control law, the switching system (11) can be stable as soon as possible and we can obtain a stable output voltage. We will prove the feasibility of this switching law in the following theorem.

**Theorem** **2.** Let $p_{11},p_{22}>0,\frac{p_{11}}{L}=\frac{p_{22}}{C}$, if exist $v_{in}<v_{c}^{*}\leq \frac{\sqrt{R_{load}}V_{in}}{2\sqrt{R}}$, and $i_{L}^{*}=\frac{V_{in}-\sqrt{V_{in}^{2}-\frac{4R{v_{c}^{*}}^{2}}{R_{load}}}}{2R}$, then the system (11) is asymptotically stable under the upper control law. Moreover, the system (11) has the unique equilibrium point $\left(i_{L}^{*},v_{c}^{*}\right)$.

**Proof** **of Theorem 2.** For guaranteeing the stability of the system (11), we consider $\dot{V}\left(x\right)<0$. There exist two derivatives ${\dot{V}}_{0}$(x) {S = 0} and ${\dot{V}}_{1}$(x) {S = 1}.

(1) For S = 0, we have ${\dot{V}}_{0}\left(x\right)<0$. Substituting the parameters $V_{in},R,R_{load},L,C,x^{*}$, using $\frac{P_{11}}{L}=\frac{P_{22}}{C}$ gives
  (14) $$\begin{array}{c} R{\left(i_{L}-\frac{V_{in}+Ri_{L}^{*}-v_{c}^{*}}{2R}\right)}^{2}+\frac{1}{R_{load}}{\left(v_{c}-\frac{R_{load}\left(i_{L}^{*}+\frac{v_{c}^{*}}{R_{load}}\right)}{2}\right)}^{2}\\ >\frac{{\left(V_{in}-Ri_{L}^{*}-v_{c}^{*}\right)}^{2}-4Ri_{L}^{*}v_{c}^{*}}{4R}+\frac{R_{load}{\left(i_{L}^{*}+\frac{v_{c}^{*}}{R_{load}}\right)}^{2}}{4} \end{array}$$
(2) For S = 1, we have ${\dot{V}}_{1}\left(x\right)<0$. Substituting the parameters $V_{in},R,R_{load},L,C,x^{*}$, using again $\frac{P_{11}}{L}=\frac{P_{22}}{C}$ gives
  (15) $$\begin{array}{c} R{\left(i_{L}-\frac{V_{in}+Ri_{L}^{*}}{2R}\right)}^{2}+\frac{{\left(v_{c}-\frac{v_{c}^{*}}{2}\right)}^{2}}{R_{load}}>\frac{{\left(V_{in}-Ri_{L}^{*}\right)}^{2}}{4R}+\frac{{v_{c}^{*}}^{2}}{4R_{load}} \end{array}$$

This gives the expressions

(16) $$\begin{array}{c} \Phi_{0}=\{\left(i_{L},v_{c}\right)\in R^{2}:R{\left(i_{L}-\frac{V_{in}+Ri_{L}^{*}-v_{c}^{*}}{2R}\right)}^{2}+\frac{1}{R_{load}}{\left(v_{c}-\frac{R_{load}\left(i_{L}^{*}+\frac{v_{c}^{*}}{R_{load}}\right)}{2}\right)}^{2}\\ >\frac{\left(V_{in}-Ri_{L}^{*}-v_{c}^{*}\right)-4Ri_{L}^{*}v_{c}^{*}}{4R}+\frac{R_{load}{\left(i_{2}^{*}+\frac{v_{c}^{*}}{R_{load}}\right)}^{2}}{4}\} \end{array}$$

(17) $$\begin{array}{c} \Phi_{1}=\left\{\left(i_{L},v_{c}\right)\in R^{2}:R{\left(i_{L}-\frac{V_{in}+Ri_{L}^{*}}{2R}\right)}^{2}+\frac{{\left(v_{c}-\frac{v_{c}^{*}}{2}\right)}^{2}}{R_{load}}>\frac{{\left(V_{in}-Ri_{L}^{*}\right)}^{2}}{4R}+\frac{{v_{c}^{*}}^{2}}{4R_{load}}\right\} \end{array}$$

**Figure 3:**
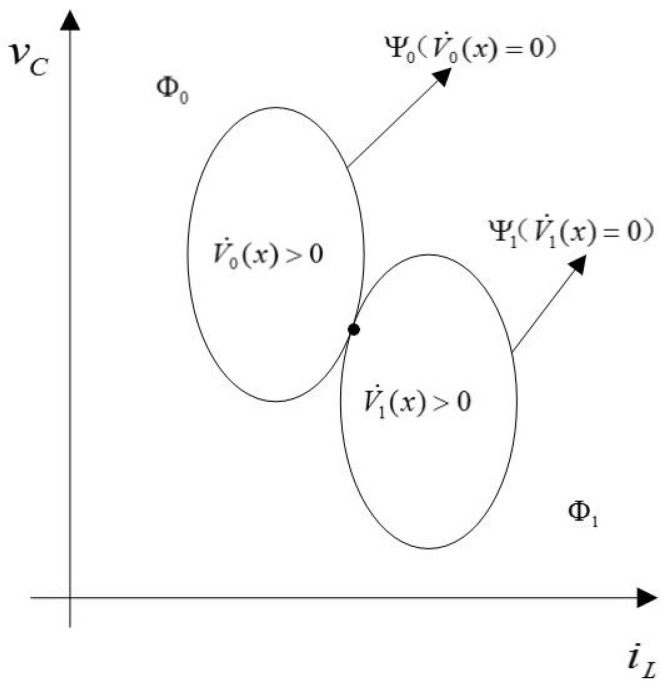
An example of a possible sign distribution for the two ellipses ${\dot{V}}_{0}\left(x\right)=0$ and ${\dot{V}}_{1}\left(x\right)=0$.

We also define $\Psi_{S}$:=$\left\{x\in R^{2}:{\dot{V}}_{S}=0\right\}$. Obviously, $\Psi_{0}$ and $\Psi_{1}$ are both ellipses. Compare $\frac{1}{R}$ to $R_{load}$, we can get the graph as in Figure 3. The graph indicates that $\Phi_{0}$ is the external area of the ellipse $\Psi_{0}$. $\Phi_{1}$ is the external area of the ellipse $\Psi_{1}$. For guaranteeing system (11) is asymptotically stable, for each $x\in R^{2}$, there exist S∈{0,1} such that $V_{S}\left(x\right)<0$. As a result, the two ellipses must have a unique crossover point at most, which leads to

(18) $$\begin{array}{c} {\dot{V}}_{0}\left(x\right)={\dot{V}}_{1}\left(x\right) \end{array}$$

we have

(19) $$\begin{array}{c} \left(\frac{R{i_{L}^{*}}^{2}}{v_{c}^{*}}+\frac{1}{R_{load}}\right){v_{c}}^{2}-\left(\frac{i_{L}^{*}V_{in}}{v_{c}^{*}}+\frac{R{i_{L}^{*}}^{2}}{v_{c}^{*}}+\frac{v_{c}^{*}}{R_{load}}\right)v_{c}+V_{in}i_{L}^{*}=0 \end{array}$$

Then Equation (19) should have a unique solution at most. By solving (19), we can get

(20) $$\left\{\begin{array}{ll} i_{L}^{*} & =\frac{V_{in}-\sqrt{V_{in}^{2}-\frac{4R{v_{c}^{*}}^{2}}{R_{load}}}}{2R}\\ v_{c}^{*} & \leq \frac{\sqrt{R_{load}}V_{in}}{2\sqrt{R}} \end{array}\right.$$

Moreover, we can obtain $\left(i_{L}^{*},v_{c}^{*}\right)$ is the unique crossover point of two ellipses which is the equilibrium point of system (11). The proof of the theorem is complete. ☐

**Remark** **4.** $\left(i_{L}^{*},v_{c}^{*}\right)$ actually is the intersection point of two ellipses. It is also a equilibrium point of the switching law. In order to ensure that the equilibrium point is unique, the intersection point of two ellipses is only one. Then we can get $i_{L}^{*}=\frac{V_{in}-\sqrt{V_{in}^{2}-\frac{4R{v_{c}^{*}}^{2}}{R_{load}}}}{2R}$ and $v_{c}^{*}\leq \frac{\sqrt{R_{load}}V_{in}}{2\sqrt{R}}$ from the process of solving Equation (19).

### 4.2. Separation Principle

In order to realize system reconfiguration, state value *x* and output value *y* are replaced by $\hat{x}$ and $x^{*}$. Therefore, we will discuss the feasibility of this system reconfiguration approach. Let $\vartheta \left(t\right)=\left[\begin{array}{c} e(t)\\ z(t) \end{array}\right].$ Since $e\left(t\right)=x\left(t\right)-\hat{x}\left(t\right),z\left(t\right)=x\left(t\right)-x^{*}$ it has

(21) $$\begin{array}{c} \dot{\vartheta }\left(t\right)=\left[\begin{array}{c} \dot{e}\left(t\right)\\ \dot{z}\left(t\right) \end{array}\right]=\left[\begin{array}{cc} A_{\sigma }-HC & 0\\ 0 & A_{\sigma } \end{array}\right]\left[\begin{array}{c} e(t)\\ z(t) \end{array}\right]+\left[\begin{array}{c} 0\\ A_{\sigma }x^{*}+Bu\left(t\right) \end{array}\right] \end{array}$$

Calculating the poles of augmented system (21), then there

(22) $$\begin{array}{cc} det[sI-\left[\begin{array}{cc} A_{\sigma }-HC & 0\\ 0 & A_{\sigma } \end{array}\right]] & =det\left[\begin{array}{cc} sI-(A_{\sigma }-HC) & 0\\ 0 & sI-A_{\sigma } \end{array}\right]\\ & =det\left[sI-\left(A_{\sigma }-HC\right)\right]\cdot det\left[sI-A_{\sigma }\right] \end{array}$$

After the system reconfigured, it has

$$\begin{array}{cc} \dot{\hat{x}}\left(t\right) & =A_{\sigma }\hat{x}\left(t\right)+Bu+H\left(x^{*}-\hat{y}\left(t\right)\right)\\ \hat{y}\left(t\right) & =C\hat{x}\left(t\right) \end{array}$$

There exist $e\left(t\right)=x\left(t\right)-\hat{x}\left(t\right)$ and $z^{*}\left(t\right)=\hat{x}\left(t\right)-x^{*}$, it has

$$\begin{array}{cc} \dot{e}\left(t\right) & =A_{\sigma }e\left(t\right)+Hz^{*}\left(t\right)\\ {\dot{z}}^{*}\left(t\right) & =\left(A_{\sigma }-HC\right)z^{*}\left(t\right)+\left(A_{\sigma }x^{*}+Bu\left(t\right)\right) \end{array}$$

Denoting $\eta \left(t\right)=\left[\begin{array}{c} e(t)\\ z^{*}\left(t\right) \end{array}\right]$ and calculating the poles

(23) $$\begin{array}{cc} det[sI-\left[\begin{array}{cc} A_{\sigma } & H\\ 0 & A_{\sigma }-HC \end{array}\right]] & =det\left[\begin{array}{cc} sI-A_{\sigma } & -H\\ 0 & sI-(A_{\sigma }-HC) \end{array}\right]\\ & =det\left[sI-A_{\sigma }\right]\cdot det\left[sI-\left(A_{\sigma }-HC\right)\right] \end{array}$$

Separation principle is applied in this process. From Equations (22) and (23) , we can see that the poles of ϑ(t) and η(t) are the same. It indicates that ϑ(t) and η(t) are independent of each other. Thus, the designs of the Luenberger observer and the switching control law can be independent. Hence the system reconfiguration approach is feasible.

## 5. Fault Detection And System Reconfiguration Scheme Design

### 5.1. Residual Generation and Threshold Calculation

We calculate the threshold of sensor faults in this section. In this paper, we take into account observerd errors and measurement noise when calculating the threshold. We use residual evaluation function to calculate the threshold. This can minimize misdiagnosis. This paper focuses on three kinds of sensor faults in current sensor and voltage sensor, including open-circuit fault, gain deviation fault, and noise abnormity fault. In this paper, the designed Luenberger state-observer is used to detect three kinds of sensor faults.

In sensor fault detection, the measured value and observed value can produce residuals. The residual error for current of inductor and voltage of resistor are determined to be

(24) $$\left\{\begin{array}{cc} r_{i}\left(t\right) & =i_{L}\left(t\right)-{\hat{i}}_{L}\left(t\right)\\ r_{v}\left(t\right) & =V_{c}\left(t\right)-{\hat{V}}_{c}\left(t\right) \end{array}\right.$$

Define the residual $r_{1}\left(t\right)=r_{i}^{2}\left(t\right)+r_{v}^{2}\left(t\right)$. In healthy mode, the residual error maintain at pretty low value.The residual increases instantly when sensor faults happen. We need to choose a threshold to guarantee the precision of fault detection and avoid misdiagnosis. This paper adopts residual evaluation function when calculating the threshold. The threshold can be chosen as

(25) $$\begin{array}{c} J\left(r_{1}\left(t\right)\right)=\sqrt{\frac{1}{M}\int_{0}^{M}r_{1}{\left(\tau \right)}^{T}r_{1}\left(\tau \right)d\tau }\; \end{array}$$

where 0 is the initial evaluation time instant and *M* is the evaluation time step. Once the evaluation function has been selected, we are able to determine the threshold. The threshold $J_{th}$ is selected such that f(t)=0. Thus, the threshold in this paper is determined as

(26) $$J_{th}=\underset{f(t)=0}{\sup}J(r_{1}\left(t\right)).$$

Based on this, the occurrence of faults can be detected by comparing $r_{1}\left(t\right)$ and $J_{th}$ according to the following logic rule:

$$\left\{\begin{array}{c} r_{1}\left(t\right)\leq J_{th},\;\mathrm{the}\;\mathrm{system}\;\mathrm{no}\;\mathrm{alarm},\\ r_{1}\left(t\right)>J_{th},\;\mathrm{the}\;\mathrm{system}\;\mathrm{with}\;\mathrm{alarm}. \end{array}\right.$$

### 5.2. Control System Reconfiguration

Fault detection and system reconfiguration can improve the fault-tolerant ability of system operation. By means of system reconfiguration, system can operate safely and maintain stability when sensor faults happen.

System reconfiguration contains two parts in this paper. The Luenberger state-observer operates normally when sensor fault happens. Hence, the value of Luenberger observer is applied to system switching law instead of measured value. In addition, the output value *y* is replaced by the desired value $\left(i_{L}^{*},v_{c}^{*}\right)$ at the same time. Thus, fault tolerant control is realized by taking this system reconfiguration approach. This fault detection and system reconfiguration method is applied to three kinds of sensor faults in the current sensor and the voltage sensor.

## 6. Simulation Results

The efficiency of this approach is demonstrated by simulations in this section. The Boost converter model is built under MATLAB/Simulink. The simulations are performed using $V_{in}=190$ V, C=0.00285 F, L=0.005 H, $R=0.082\;\Omega ,R_{load}=100\;\Omega ,$$\left(i_{L}^{*},v_{c}^{*}\right)=\left(7.6,380\right).$ To better meet the performance index (6) and make γ get the minimum value. We give $a_{11}=299,a_{12}=152$. By solving the LMI (7), and the FD observer gain matrix is shown as:

$$\begin{array}{c} H=\left[\begin{array}{cc} 36.4677 & -17.0967\\ 28.5219 & 75.6359 \end{array}\right] \end{array}$$

In this paper, we choose the threshold as $J_{th}=0.02$. The three kinds of sensor faults in current sensor and voltage sensor are simulated when the system is in stable operation.

**Figure 4:**
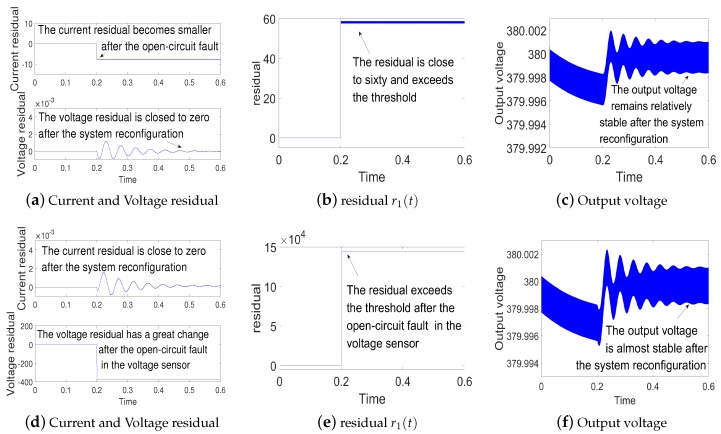
(**a**–**c**) are simulation results for the open-circuit fault in current sensor; (**d**–**f**) are simulation results for the open-circuit fault in voltage sensor.

**Figure 5:**
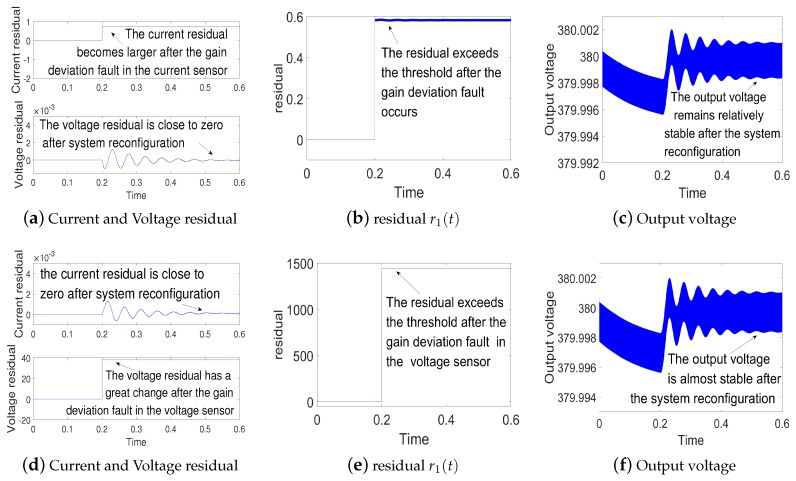
(**a**–**c**) are simulation results for current gain deviation fault in current sensor; (**d**–**f**) are simulation results for voltage gain deviation fault in voltage sensor.

**Figure 6:**
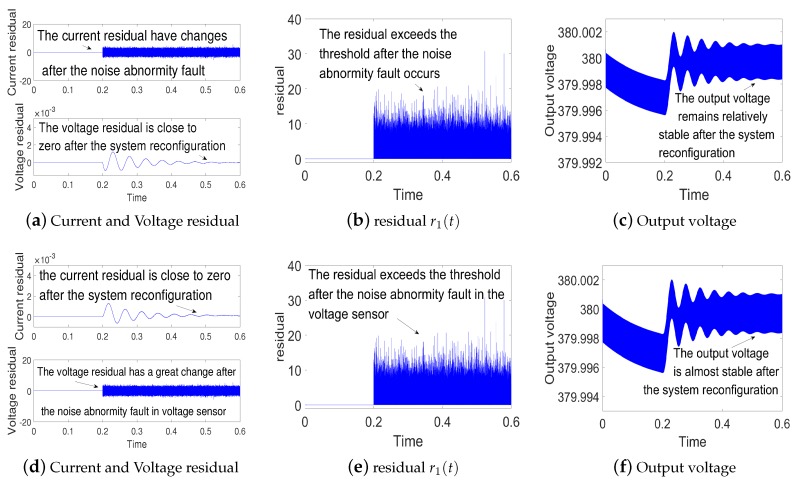
(**a**–**c**) are simulation results for current noise abnormity in current sensor; (**d**–**f**) are simulation results for voltage noise abnormity in voltage sensor.

(1) Simulations for open-circuit fault in current sensor and voltage sensor: The simulation results for the open-circuit fault are shown in Figure 4. The state-observer still works properly when open-circuit fault occurs. Since the open-circuit fault occurs at t=0.2 s, the residual error $r_{1}\left(t\right)$ is larger than the threshold. Hence the measured values are replaced by the observed values to realize system reconfiguration. According to Figure 4c,f, the output voltage remains relatively stable after the open-circuit fault. This indicates fault tolerant control is realized.
(2) Simulations for gain deviation fault in current sensor and voltage sensor: the gain deviation fault occurs separately in current sensor and voltage sensor at t=0.2 s. The simulation results are shown in Figure 5. As shown in Figure 5b,e, the residual error $r_{1}\left(t\right)$ increases and oversteps the threshold, which indicates the current sensor fault and voltage sensor fault. Then the system is reconfigured. Finally, we can get a relatively stable output voltage.
(3) Simulations for noise abnormity fault in current sensor and voltage sensor:a group of random noise is added separately on the measured values of the current $i_{L}$ and the voltage $v_{c}$ at t=0.2 s. The simulation results are shown in Figure 6. As Figure 6b,e suggests, the residual error $r_{1}\left(t\right)$ keeps to zero before t=0.2 s. Then the residual error $r_{1}\left(t\right)$ increases and overtakes the threshold, which indicates noise abnormity fault in current sensor and voltage sensor. By the fault tolerant control, we can get a relatively stable output voltage.

**Remark** **5.** *The above simulation results validate the effectiveness of the proposed metnod in the Boost converter. Besides, we applied this method to converters, including Buck converters and Buck-Boost converters. In addition, the validity of the presented approach is demonstrated by simulation examples.*

## 7. Conclusions

This fault detection and system reconfiguration method is applied to three kinds of sensor faults in the current sensor and the voltage sensor. The simulation results are shown to test and verify the effectiveness of the approach. The system is able to maintain stability by taking system reconfiguration approach. We can get a relatively stable output voltage after occurrences of sensor faults. We will build a experimental platform to verify the proposed fault detection and system reconfiguration method in the follow-up work.

## Abbreviations

The following abbreviations are used in this manuscript:

AC	Alternating current
DC	Direct current
